# A Multi-Scale Approach to Investigating the Red-Crowned Crane–Habitat Relationship in the Yellow River Delta Nature Reserve, China: Implications for Conservation

**DOI:** 10.1371/journal.pone.0129833

**Published:** 2015-06-11

**Authors:** Mingchang Cao, Haigen Xu, Zhifang Le, Mingchang Zhu, Yun Cao

**Affiliations:** 1 Nanjing Institute of Environmental Sciences, Ministry of Environmental Protection, Nanjing City, Jiangsu Province, China; 2 Hubei University, Wuhan City, Hubei Province, China; Institute of Zoology, CHINA

## Abstract

The red-crowned crane (*Grus japonensis* (Statius Müller, 1776)) is a rare and endangered species that lives in wetlands. In this study, we used variance partitioning and hierarchical partitioning methods to explore the red-crowned crane–habitat relationship at multiple scales in the Yellow River Delta Nature Reserve (YRDNR). In addition, we used habitat modeling to identify the cranes’ habitat distribution pattern and protection gaps in the YRDNR. The variance partitioning results showed that habitat variables accounted for a substantially larger total and pure variation in crane occupancy than the variation accounted for by spatial variables at the first level. Landscape factors had the largest total (45.13%) and independent effects (17.42%) at the second level. The hierarchical partitioning results showed that the percentage of seepweed tidal flats were the main limiting factor at the landscape scale. Vegetation coverage contributed the greatest independent explanatory power at the plot scale, and patch area was the predominant factor at the patch scale. Our habitat modeling results showed that crane suitable habitat covered more than 26% of the reserve area and that there remained a large protection gap with an area of 20,455 ha, which accounted for 69.51% of the total suitable habitat of cranes. Our study indicates that landscape and plot factors make a relatively large contribution to crane occupancy and that the focus of conservation effects should be directed toward landscape- and plot-level factors by enhancing the protection of seepweed tidal flats, tamarisk-seepweed tidal flats, reed marshes and other natural wetlands. We propose that efforts should be made to strengthen wetland restoration, adjust functional zoning maps, and improve the management of human disturbance in the YRDNR.

## Introduction

The importance of scale for understanding ecological patterns and processes is widely recognized [1]. Multi-scale approaches can potentially describe bird—habitat relationships more accurately than single-scale approaches because avian habitat use is generally thought to be a hierarchical process involving a range of organizational levels [2–4]. Studies at a single arbitrarily chosen spatial scale may overlook bird—habitat relationships at finer or coarser scales [5–7]. In addition, cross-scale correlations (multicollinearity among predictors at different scales) have the potential to lead to spurious conclusions regarding bird habitat use at any scale [8]. Variation partitioning and hierarchical partitioning methods have been shown to be useful tools that avoid these problems [9–15]. These methods can examine species—environment relationships by decomposing the variation in response variables into independent components that reflect the relative importance of individual predictors or groups of predictors and their joint effects [16]. This information can assist local managers in implementing specific conservation and management efforts for rare and endangered birds.

The red-crowned crane (*Grus japonensis*) is one of the most endangered waterbirds in the world, and the global wild population is estimated to be approximately 2800 [17]. The species is listed as endangered by the International Union for Conservation of Nature (IUCN) [18] due to its rarity. The red-crowned crane has two separate populations: one island population and one continental population. The island population is non-migratory and lives in southeastern and northeastern Hokkaido, Japan. The continental population is migratory, breeding mainly in Northeast China and far Southeast Russia and wintering on the Korean Peninsula and in the eastern coastal wetlands of China [17]. The red-crowned crane is a wetland specialist that prefers reed marsh and intertidal mudflat habitats, both of which have low vegetation cover, shallow water, abundant food and low levels of human disturbance [19–20]. However, in recent years, many cranes have been forced to change their habitat from natural grasslands to artificial wetlands (e.g., farmland, fish ponds, and rice fields) due to the loss and deterioration of natural wetlands throughout its range (including breeding, stopover, and wintering grounds) caused by increasing human disturbance [17, 21–22].

The Yellow River Delta Nature Reserve (YRDNR) is located in the middle of the East Asia-Australasian Flyway and is a key stopover site for the red-crowned crane and other migrating waterbirds [23]. In the last 50 years, the wetland ecosystems of the YRDNR have undergone dramatic alterations. These changes have largely resulted from the frequent low-flow conditions of the Yellow River and increases in oil exploration, road construction, and marsh reclamation [24–25]. The amounts of runoff and sediment discharge from the Yellow River have decreased considerably since the 1950s, and it is believed that the frequent and prolonged zero-flow conditions of the lower reaches of the Yellow River since the 1970s have been a major contributor to the degradation of both natural wetlands and bird habitats [24, 26]. However, this condition has improved considerably since the initiation of a wetland restoration project by the YRDNR in 2002 [26–27]. The YRDNR is also one of the most important petroleum production basements in China, where the country's second largest oil field (Shengli oil field) has been located since the early 1960s [25]. These surges in oil exploration, road construction, and marsh reclamation in recent years have undoubtedly exacerbated wetland degradation, leading to substantial habitat loss and fragmentation of the red-crowned crane population [24, 26].

Although numerous studies have examined habitat use, habitat change, and habitat suitability of red-crowned cranes in the YRDNR [20, 24, 26], an integrated multi-scale analysis of the crane—habitat relationship is lacking, and no study has considered the issue of cross-scale correlations among predictors to identify the independent and joint effects of habitat factors on the red-crowned crane. In this paper, we adopt variation partitioning and hierarchical partitioning to explore the crane—habitat relationship in the YRDNR at multiple spatial scales. The goals of the present study are to (1) determine the relative importance of spatial and habitat (plot, patch, landscape) factors on red-crowned crane occurrence; (2) investigate the unique and joint effects of plot, patch, and landscape factors on habitat use by the red-crowned crane; and (3) generate a habitat-suitability map of the red-crowned crane and compare this map with the functional zoning map of the reserve to identify protection gaps in the YRDNR.

## Methods

### Ethics statement

Our field surveys were authorized by the Management Bureau of the Yellow River Delta Nature Reserve of China. This research was conducted in strict accordance with animal care permits issued by China’s State Forestry Administration. No further specific permissions were required for our study. Our field surveys did not require permits from an institutional animal care and use committee (IACUC) or equivalent animal ethics committee because there was no physical sampling or other potentially harmful activities toward the birds. In our fieldwork, a telescope was used to observe the red-crowned crane occurrences from a minimum distance of 500 m to minimize disturbance. Care was to taken to minimize the negative impact on red-crowned cranes during the periods of habitat surveying at the plot scale.

### Study area

**Fig 1:**
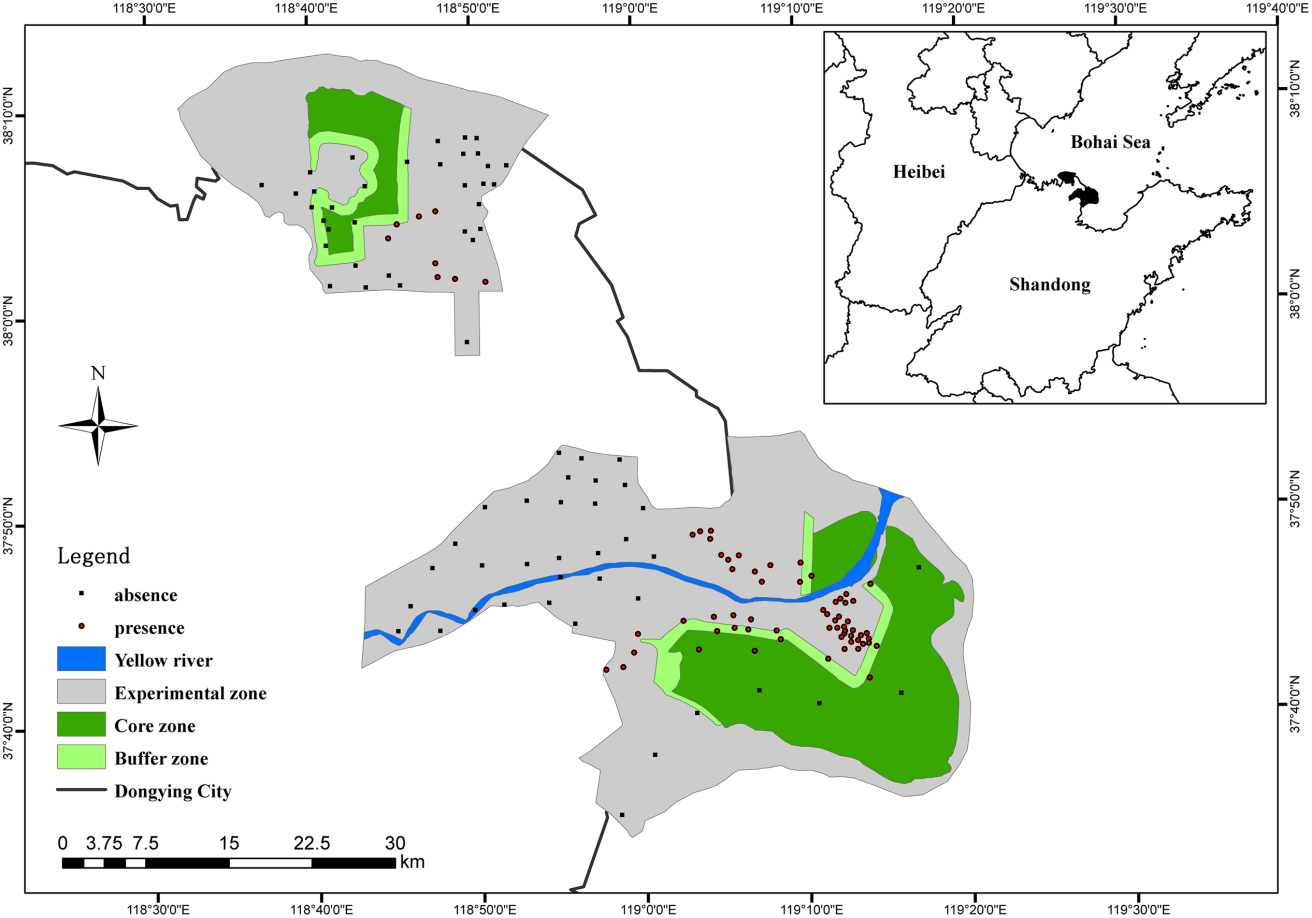
Location of the Yellow River Delta Nature Reserve in China. 'Absence' and 'presence' indicate the absence and presence sampling points of red-crowned cranes. Blue: Yellow River in the YRDNR. Light gray: experimental zones of the YRDNR. Light green: buffer zones of the YRDNR. Dark green: core zones of the YRDNR.

The YRDNR covers an area of 153,000 ha and is located in the Yellow River estuary in northeastern Shandong Province, China (37°35′–38°12′N, 118°33′–119°20′E) (Fig 1). The YRDNR is a national nature reserve that was established in 1992 to protect the new wetlands at the mouth of the Yellow River and rare and endangered birds. The abundance of tidal flats and marshes, wetland vegetation and aquatic organisms within the YRDNR provides a highly suitable habitat for waterbird survival, reproduction and migration. Furthermore, the YRDNR is a site in the East Asia bird migration network and the East Asia-Australia wading-bird network [28]. However, the Shengli oil field, which is the second largest oil field in China, is also located in the YRDNR and poses a threat to the waterbird habitat.

### Red-crowned crane surveys

Surveys were conducted from October to December 2007. We conducted several regular route surveys by vehicle or on foot between 8:00 A.M. and 4:00 P.M. during suitable weather conditions. To avoid replicated sampling, surveys were not conducted more than once per day. Binoculars (8×42) were used to detect red-crowned cranes along the transect. When cranes were observed or their loud calls were heard, provided that no impassable tidal channels or river barriers existed, we approached the birds and used GPS to accurately record their location. We then sampled vegetation and water variables at the plot scale. Because the red-crowned crane is susceptible to human disturbance and difficult to track, we also located birds by identifying their footprints during the surveys. Based on these techniques, 68 occurrence points were recorded.

Random absence points (70) were recorded for locations where cranes were not detected during at least two surveys. To maximize the probability of absence, we sampled each random point at a distance at least 3 km from the nearest presence site, as the cranes’ maximum movement distance is approximately 3 km [29].

At each sampling site, we measured vegetation structure within a 30-m radius of the survey point. We established 1 square area (1×1 m) at the center and 2 square areas (1×1 m) in each direction to the north, east, south and west within the circular area. In each square, we recorded the following variables: (1) vegetation structure; specifically, the maximum heights and coverages of tamarisk (*Tamarix chinensis*), seepweed (*Suaeda heteroptera*), reed (*Phragmites communis*) and other plants; and (2) water variables; specifically, water area and water depth. We then calculated the average values for each vegetation type and for the water variables across the 9 squares.

### Landscape and spatial data

#### Data sources

We extracted maps of vegetation, water and human disturbance from remote-sensing images within the YRDNR. We used a TM image and SPOT-5 image in this study. The TM image was from October 2, 2006, and the SPOT-5 image (spatial resolution 2.5 m) was from September 7, 2005.

#### Vegetation map

We combined supervised classifications, unsupervised classifications and visual interpretations to generate a vegetation map using the rectified SPOT-5 remote-sensing image. The main vegetation types included woodland, Chinese tamarisk shrub, reed meadow, reed marsh, seepweed tidal flat, Chinese tamarisk-seepweed tidal flat, bare tidal flat, salt pan, shrimp pond, deep-water area and farmland.

#### Water resources and human disturbance map

We conducted supervised and unsupervised classifications to obtain a water-resources map of the YRDNR using the TM 5/7-band image, which is very sensitive to water features. We combined the SPOT-5 image with 1:50,000 topographic maps to extract roads, oil wells and other human disturbance factors.

#### Patch- and landscape-level data

We used FRAGSTATS 4.0 to extract patch- and landscape-level data from the vegetation, water and human disturbance maps based on 138 sampling points [30].

#### Patch-level data set

The patch-level data set was constructed with a set of five variables that were related to the structure of the habitat patch in which each sampling point was located. Patch variables were obtained from the vegetation map using FRAGSTATS analysis and consisted of Patch area (AREA), Shape index (SHAPE), Core area (CORE), Edge contrast index (ECON), and Euclidean nearest-neighbor distance (ENN).

#### Landscape-level data set

The landscape-level data set included landscape composition and configuration metrics at twelve spatial scales. We used the moving-window function in FRAGSTATS 4.0 to calculate the composition and configuration metrics for each sample point. Fourteen configuration metrics and composition indices for each vegetation type, water source, road density, and oil-well density were generated at each of the twelve spatial scales by shifting the moving-window size (10, 50, 100, 200, 350, 500, 750, 1000, 1250, 1500, 1750, and 2000 ha).

#### Spatial data set

The spatial data set consisted of seven spatial variables constructed from the xy coordinates of each plot site. First, the xy coordinates of the plot sites were centered on their means [31]. Next, five higher and cross-product terms were calculated (xy, x^2^, y^2^, x^2^y, y^2^x) on the xy coordinates. Finally, each variable was divided by its standard deviation.

### Variable selection

To avoid multicollinearity, we first performed Spearman (two-sided) correlation analyses between any two variables within the plot, patch and spatial data sets. If the correlation coefficient between any two variables was >0.7, we retained the variable that explained the greatest deviance for analysis in univariate logistic models. For example, at the plot scale, reed height and reed coverage were highly correlated (r = 0.934); therefore, we excluded reed height from further analysis because the explained deviance of reed coverage in the univariate model was greater than that of reed height. Next, we performed forward stepwise regression to select the variables that were significant at p<0.05, resulting in final plot, patch and spatial data sets that consisted of six plot variables, four patch variables and three spatial variables, respectively (Table 1).

**Table 1:** Descriptions of the habitat variables at the plot, patch and landscape scales.

Scale	Variable name	Code	Descriptions of variables
Plot **variables**	Tamarisk coverage	TC	Average coverage of tamarisk within a 30-m radius circular area
	Reed coverage	RC	Average coverage of reed within a 30-m radius circular area
	Seepweed coverage	SC	Average coverage of seepweed within a 30-m radius circular area
	Vegetation coverage	VC	Average coverage of vegetation within a 30-m radius circular area
	Maximum vegetation height	MVH	Maximum height of vegetation within a 30-m radius circular area
	Water depth	WC	Average water depth within a 30-m radius circular area
Patch **variables**	Patch area	AREA	Patch area of sampling points
	Patch shape index	SHAPE	Patch shape index of sampling points
	Patch distance	ENN	Euclidean nearest-neighbor distance of sampling points
	Patch edge	ECON	Patch edge contrast index of sampling points
Landscape **variables**	Percentage of reed marshes	RMP	Percentage of reed marshes at a 10-ha scale
	Percentage of tamarisk-seepweed tidal flats	TSTP	Percentage of tamarisk-seepweed tidal flats at a 10-ha scale
	Percentage of seepweed tidal flats	STP	Percentage of seepweed tidal flats at a 50-ha scale
	Road density	RDD	Road density at a 100-ha scale
spatial **variables**	x, x^2^, x^2^y	-	The x coordinates, its quadratic term, and cross-product term with y at each sampling point

For the landscape-scale analysis, we first performed univariate logistic models for each metric at the twelve spatial scales to select the scale that explained the greatest deviance in the twelve models. Second, we performed forward stepwise regression on the selected scales of all the landscape metrics. Third, the remaining landscape variables were entered into the same screening procedure at the plot, patch and spatial scales. We finally obtained four landscape metrics: the percentage of seepweed tidal flat (STP) at the 50-ha scale, the percentage of tamarisk-seepweed tidal flat (TSTP) at the 10-ha scale, the percentage of reed marsh (RMP) at the 10-ha scale, and road density (RDD) at the 100-ha scale (Table 1).

### Statistical analysis

#### Variance partitioning

**Fig 2:**
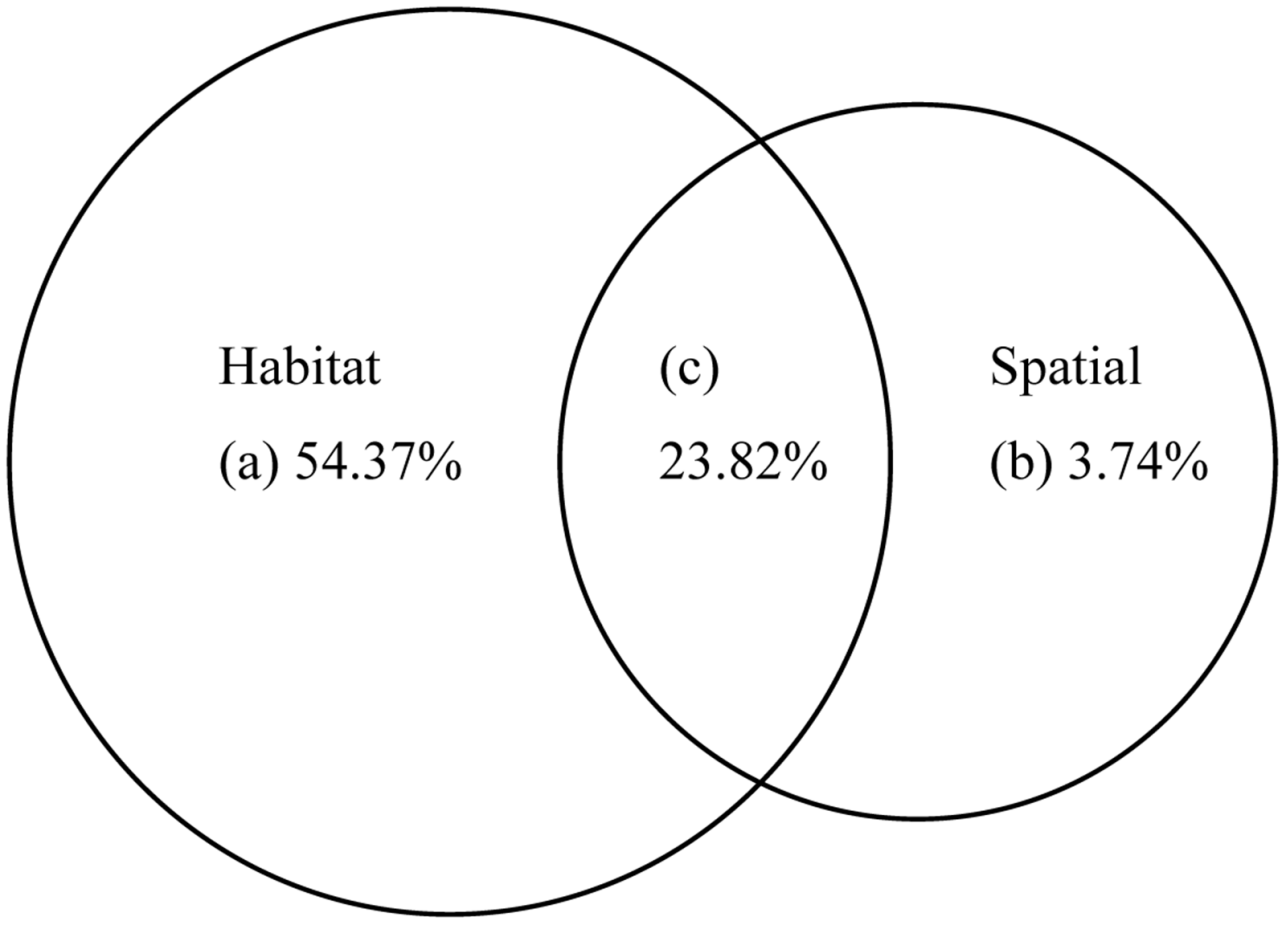
Percentages of the total variance in red-crowned crane occurrence data explained by habitat and spatial variables at the first hierarchical level. (a) The variance explained by habitat variables; (b) the variance explained by spatial variables; and (c) the variance explained by combination of habitat and spatial variables.

**Fig 3:**
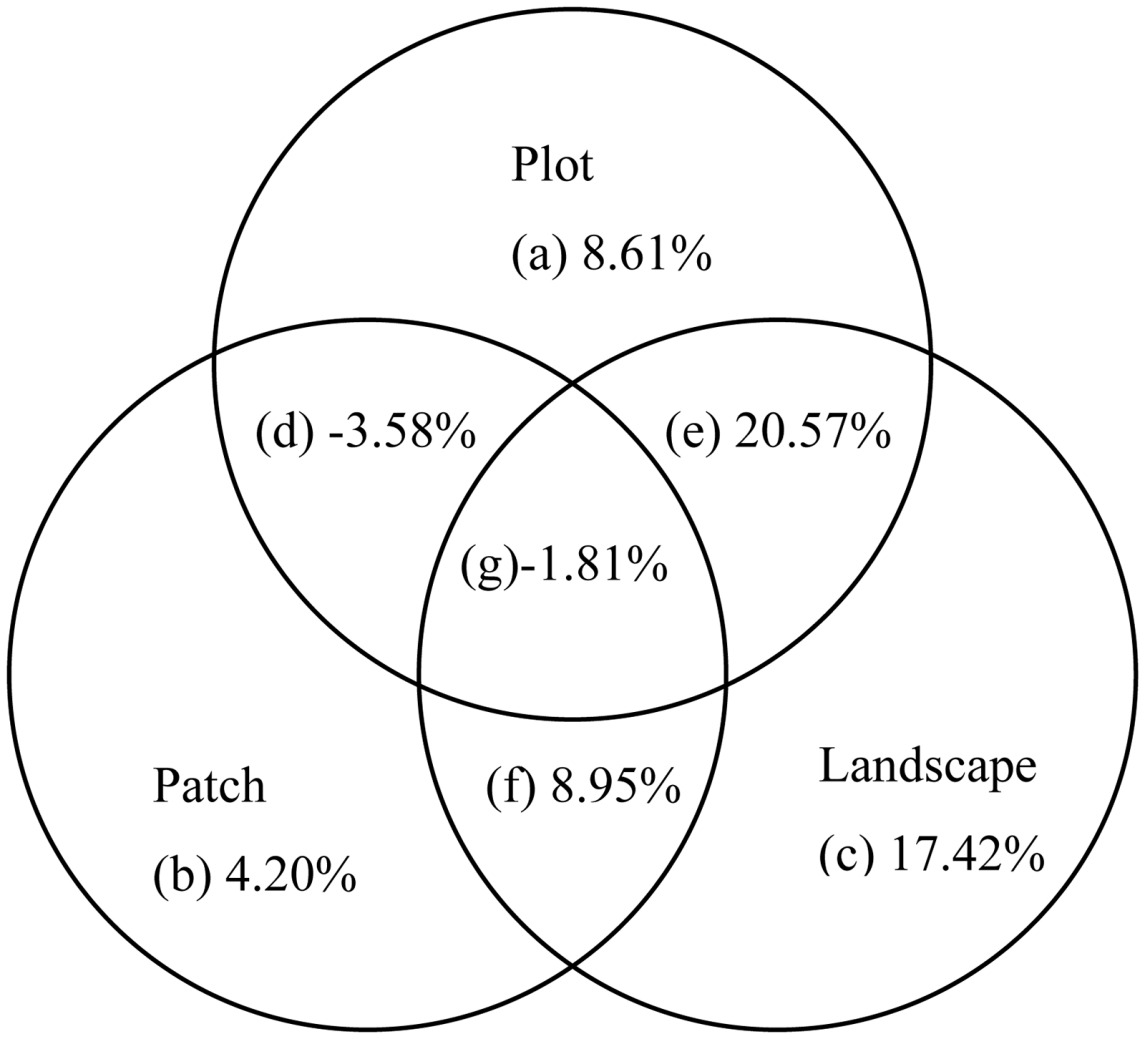
Percentages of the total variance in red-crowned crane occurrence data explained by plot, patch, and landscape variables at the second hierarchical level; a, b, c are unique effects of plot, patch and landscape variables, respectively; d, e, f, g are fractions indicating their combined effects.

Variance partitioning is a quantitative statistical method by which the variation in dependent variables can be decomposed into independent components reflecting the relative importance of different groups of explanatory variables and their joint effects [7, 9]. In this study, variance partitioning was used to decompose the explained variance of the red-crowned crane occurrence data into independent and joint components at two hierarchical levels. The first level of the decomposition was conducted between spatial variables and habitat variables (plot, patch and landscape data sets combined) (Fig 2). At the second level of decomposition, the habitat variables were partitioned into three groups of explanatory variables at the plot, patch and landscape scales. In this analysis, spatial variables were considered as covariables to remove their effects. A series of (partial) binomial logistic regression models were used to calculate the variance decomposition values at the first and second levels using R 2.15 software [32]. This procedure resulted in three fractions at the first level: pure spatial effects, the joint effects of spatial and habitat variables, and pure habitat effects. The second level was decomposed into seven fractions: (a) pure plot-level effects, (b) pure patch-level effects, (c) pure landscape-level effects, combined variation due to the joint effects of (d)plot and patch variables, (e) plot and landscape variables, (f) patch and landscape variables, and (g) plot, patch and landscape variables [11] (Fig 3).

#### Hierarchical partitioning

We used hierarchical partitioning to determine the independent contribution of habitat variables at each scale to the red-crowned crane occurrence data [33]. The statistical significance of the independent effects of each variable was tested using a 1000-randomization procedure [34]. The result of each significance test was expressed as a z-score. A z-score greater than or equal to 1.65 was considered statistically significant at p<0.05. Because hierarchical partitioning requires models with no more than 12 predictor variables, we used plot, patch and landscape variables separately to conduct hierarchical partitioning. We conducted this analysis with the ‘hier.part’ package implemented in R 2.15 software [32, 35]. Because hierarchical partitioning did not identify whether predictor variables have a positive or negative relationship with red-crowned crane occurrence, we adopted the final forward stepwise regression results in the variable-selection approach to interpret the results.

#### Habitat model

We used a binomial logistic regression model to relate red-crowned crane occurrence to the selected four landscape variables. Because spatial autocorrelation was found in both the response variable and in the residuals of our model’s data set, we built an autologistic model by including an autocovariate variable in the logistic regression model, following Augustin et al. [36]. The autocovariate term allowed us to measure the spatial autocorrelation in the response variable. We used the ‘spdep’ package in R 2.15 software to calculate autocovariates at neighborhood sizes from 0 to 20 km at 2-km intervals [32, 37]. The model’s autocovariate term was calculated as follows:

$$auto{\text{cov}}_{i}=\frac{\sum_{j=1}^{k_{i}}w_{ij}y_{j}}{\sum_{j=1}^{k_{i}}w_{ij}}$$

where w_ij_ is the inverse of the Euclidean distance between sample points *i* and *j* and *y* represents the response variable. The model’s AIC value was used to identify the neighborhood size that produced the best autologistic model.

A 10-fold cross-validation was applied to test the model’s accuracy. We used the ‘PresenceAbsence’ package in R 2.15 software to evaluate the discrimination ability of the final and cross-validated models by estimating the sensitivity, specificity, correct classification rate and Cohen’s kappa [32, 38–39]. The classification thresholds were established by maximizing the kappa values. We also calculated the area under the receiver operating characteristic curve (ROC, AUC), which is a threshold-independent measure [40].

We used the autologistic model to obtain a predicted probability map that shows the probability of red-crowned crane occurrence within each pixel of the study area. We used the classification threshold that maximized the kappa values to convert probability values to habitat suitability values of 0 (unsuitable habitat) or 1 (suitable habitat). Based on the habitat suitability map, we used FRAGSTATS analysis to calculate the related landscape indices that reflect the habitat quality of the red-crowned crane in the YRDNR, including the total suitable habitat area (CA), proportion of suitable habitat area (PLAND), and average patch area of suitable habitat (AREA_MN) [30]. We also calculated the above landscape indices separately for the northern and southern parts of the YRDNR to examine regional variation. Finally, we overlaid the habitat suitability map with the YRDNR functional zoning map to identify the protection gaps for red-crowned cranes. A protection gap was considered present where crane suitable habitat fell outside the core zone of the reserve.

## Results

### Hierarchical variance partitioning

At the first hierarchical level, spatial and habitat variables in total explained 81.93% of the variation in the occurrence data. The independent effect of habitat variables (54.37%) was significantly larger than that of the spatial variables (3.74%) and the joint effect of spatial and habitat variables (23.82%) (Fig 2).

At the second hierarchical level, landscape-level factors had both the largest total effect (45.13%, including joint and independent effects) and the largest independent effect on the red-crowned crane occurrence data (17.42%) (excluding spatial effects) (Fig 3). Patch variables explained the least amount of the total (7.76%) and independent (4.20%) variation in the crane occurrence data (Fig 3). In addition, the confounded variation between plot and landscape variables (20.57%) explained the largest proportion of the crane occurrence data (Fig 3).

### Hierarchical partitioning

Vegetation coverage (41.84%) at the plot scale was responsible for the largest negative independent contributions of the plot factors to the occupancy of red-crowned cranes, whereas the other four vegetation variables were responsible for significantly lower positive independent contributions. Water depth was the only plot variable for which the independent effects did not reach significance (Table 2). At the patch scale, the independent contributions of AREA (63.58%) and SHAPE (19.32%) were statistically significant and notably higher than those of the other two patch variables (ECON and ENN). All patch factors exhibited negative independent effects on red-crowned crane occurrence (Table 2). The independent effects of all landscape-level factors were statistically significant. Among the landscape factors, STP (63.07%) had the largest positive independent explanatory power. RDD was the only landscape factor that showed a negative independent effect on the red-crowned crane occurrence data (Table 2).

**Table 2:** Hierarchical partitioning results at the plot, patch and landscape scales.

Scale	Variable	Independent contribution (%)	z-value
**Plot variables**	TC	8.28	4.65*
	RC	15.67	9.53*
	SC	22.89	15.43*
	VC	-41.84	25.13*
	MVH	8.16	4.68*
	WD	-3.16	1.52
**Patch** variables	AREA	-63.58	9.68*
	SHAPE	-19.32	2.37*
	ENN	-9.13	0.70
	ECON	-7.97	0.58
**Landscape** variables	RMP	10.43	9.83*
	TSTP	12.26	11.37*
	STP	63.07	55.24*
	RDD	-14.24	11.09*

A z-value greater than or equal to 1.65 was considered statistically significant at p<0.05, as denoted by *.

### Habitat model

The final autologistic model had a neighborhood size of 8 km. The AIC value of the autologistic model (43.403) was lower than that of the logistic model (68.525), but the R^2^ (0.836) exhibited the opposite trend (Table 3), which suggests that the autologistic model has a better fit than the logistic model.

**Table 3:** Correct classification rate (CCR), sensitivity, specificity, kappa, and AUC for the final logistic model and autologistic model with 10-fold cross-validation (mean values with range in parentheses).

	AIC	R^2^	CCR	Sensitivity	Specificity	kappa	AUC
**logistic model**	68.525	0.694	0.870	0.866	0.873	0.739	0.970
	-	-	(0.529–0.928)	(0.529–1)	(0.071–1)	(0.07–0.855)	-
**autologistic model**	43.403	0.836	0.929	0.927	0.930	0.857	0.991
	-	-	(0.804–0.964)	(0.603–1)	(0.657–1)	(0.606–0.927)	-

According to the final autologistic model, RMP, STP and autocovariate variables were significant in the model. The coefficients of the variables indicated that RDD was negatively associated with red-crowned crane occurrence while the other four variables were positively associated (Table 4).

**Table 4:** Variable parameters for the final autologistic model with a neighborhood size of 8 km.

Variable	Coefficient	Standardized coefficient	z-value	p-value
Constant	-6.026	0	-3.628***	0.000285
RMP	0.043	2.410	3.020**	0.002528
TSTP	0.119	1.677	1.432	0.152219
STP	0.073	5.671	3.068**	0.002156
RDD	-4.389	-0.772	-0.846	0.397412
Autocovariate	6.651	5.625	3.453***	0.000555

**: p-value<0.01;

***: p-value <0.001

The model accuracy results showed that the logistic and autologistic models had good predictive capacity (CCR>0.87, kappa value>0.7, AUC value>0.97) but that the autologistic model was better than the logistic model (Table 3).

**Fig 4:**
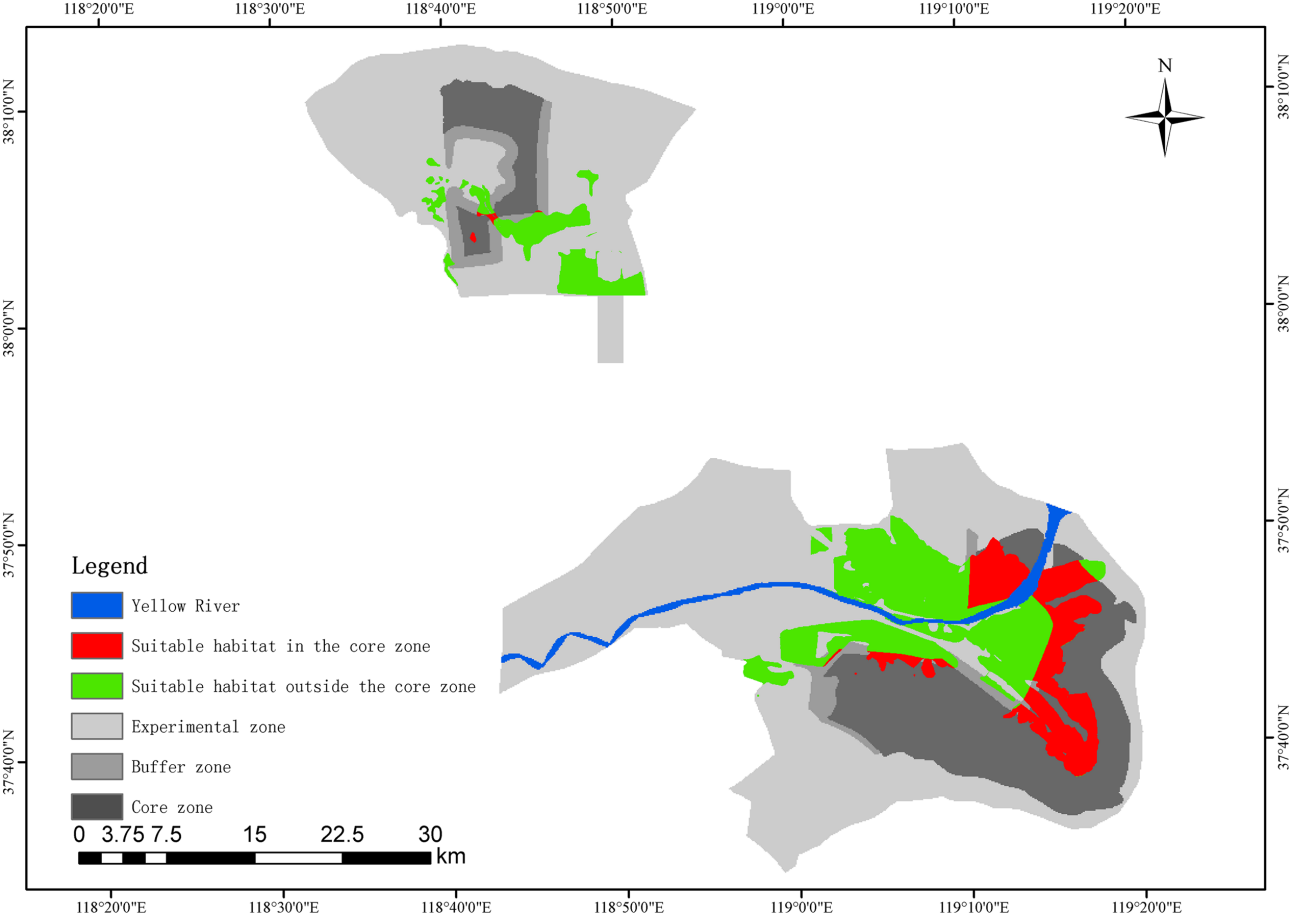
Habitat suitability map and protection gap for red-crowned cranes used by the final autologistic model. Red: suitable habitat for the red-crowned crane in the core zone of the YRDNR. Green: suitable habitat for the red-crowned crane outside the core zone of the YRDNR. Blue: Yellow River in the YRDNR. Light gray: experimental zones of the YRDNR. Gray: buffer zones of the YRDNR. Dark gray: core zones of the YRDNR.

The habitat suitability map showed that the suitable habitat for red-crowned cranes covered more than 26% of the reserve area (Table 5). Most (85%) of this suitable habitat area is located in the southern part of the reserve and along the two sides of the Yellow River estuary (Fig 4). The AREA_MEAN indicated that the southern part of the reserve had higher habitat quality than the northern part (Table 5). The gap analysis showed that the protection gap area totaled 20,455 ha (northern part: 4233 ha; southern part: 16,222 ha), accounting for 69.51% of the total suitable habitat (Table 5).

**Table 5:** The landscape index of red-crowned crane suitable habitat in the YRDNR.

Location	CA (ha)	PLAND (%)	AREA_MN (ha)	PG (ha)	PPG (%)
**North**	4333	17.90	270.78	4233	97.69
**South**	25,079	28.72	3134.88	16222	64.68
**Total**	29,412	26.32	1226.04	20455	69.51

North: northern part of the YRDNR; South: southern part of the YRDNR; Total: the entire reserve; CA: total suitable habitat area; PLAND: proportion of suitable habitat area; AREA_MN: average patch area of suitable habitat; PG: protection gap area in the YRDNR; PPG: percentage of protection gap area with total suitable habitat in the YRDNR.

## Discussion

### Factors determining red-crowned crane occurrence at multiple scales

Variation partitioning and hierarchical partitioning are proven novel statistical approaches that offer a deeper understanding of multi-scale bird-habitat relationships than traditional regression methods [11–12]. Traditional regression approaches can ignore collinearity among explanatory variables or cross-scale correlations, which could lead to distorted inferences regarding the relative importance of explanatory variables [16]. Some previous studies have employed traditional regression approaches to identify the key factors determining the habitat use of the red-crowned crane [19, 20, 41]. For example, Shu Ying et al. adopted ANOVA to investigate habitat use of the red-crowned crane in the YRDNR and found that human disturbance was the main limiting factor during the stopover period and that food was the main limiting factor during the wintering period [20]. These methods may not fully identify the relative importance of the explanatory variables because they do not provide separate measures of the amounts of variation explained independently and jointly by two or more variables or groups of variables [8, 16].

Our variation partitioning results revealed that habitat variables accounted for a larger total of independent explained variation in red-crowned crane occupancy than did spatial variables. This finding suggests that the habitat features make a dominant contribution to crane distribution. We also found that landscape factors had the highest total and independent effects on crane occupancy. This finding indicates that landscape factors have larger relative importance than plot and patch factors in influencing crane habitat use. This importance of landscape factors might result from the fact that cranes are wetland birds with a large body size and are sensitive to human disturbance. These birds may prioritize searching for suitable foraging habitat far from human activities because they live in a disturbed environment with intensive oil exploration and road development in the YRDNR [24–25]. Meanwhile, we cannot ignore the relative contribution of plot factors. The independent and total effects of these factors were significantly greater than those of patch factors. Furthermore, the combined effects between plot factors and landscape factors explained the largest variation in red-crowned crane occupancy. This finding is not unexpected, because when cranes find suitable foraging habitat, their next step may be to find a feeding site with sufficient food and little human disturbance.

Our hierarchical partitioning results found that STP had the largest independent explanatory power at the landscape scale. This finding suggests that STP is the main limiting factor for habitat use of red-crowned cranes at the landscape scale. The importance of this factor may reflect the fact that seepweed tide flats are the typical intertidal mudflats of the estuarine natural wetland located on both sides of the Yellow River estuary [42–43]. This habitat is considered an important food source (of tidal mudflat crabs) for the migratory red-crowned crane population in the YRDNR [44]. Our results also revealed that the final three selected vegetation variables (STP, RMP, and TSTP) at the landscape scale are natural habitat factors, and all reached significance. This finding confirms the results of Li et al. (2013), who found that natural habitat is a core foraging habitat for most waterbird guilds (including cranes) [43]; however, there are large differences between sites such as Yancheng Nature Reserve and the demilitarized zone of Korea, where artificial habitat (fish ponds and unplowed rise fields) was also found to provide favored habitat for red-crowned cranes [19, 22, 45].

We found that patch area had a relatively larger negative independent contribution to red-crowned crane occurrence than other factors at the patch scale. This finding suggests that red-crowned crane occurrence is negative in relation to patch area, which is in contrast with previous reports in the Yancheng Nature Reserve [22]. Three factors might explain this discrepancy. First, we compared occupied patches with unoccupied patches in our approach, whereas researchers at Yancheng compared occupied patches with all other patches in their analysis. Second, cranes might show different habitat requirements at the two sites, as the YRDNR and Yancheng are their migration site and wintering area, respectively. Third, it might be associated with the more fragmented suitable habitat occupied by cranes due to increasing human disturbance in the YRDNR [24]. Therefore, further studies are needed to determine the relative effects of the patch size of natural and artificial habitats on crane habitat use.

Food, shelter (vegetation), water and human disturbance are considered as four elements of habitat use for the red-crowned crane [46]. Our results show that the most important plot factor at the plot scale is vegetation coverage, which had the same significantly negative independent effects on crane occurrence as road density at the landscape scale. This finding is consistent with previous studies showing that cranes prefer to select foraging sites with low vegetation cover and little human disturbance [20]. This preference might exist because cranes are large wading birds with body lengths greater than 120 cm, for which denser vegetation coverage might hinder finding food and thereby reduce foraging efficiency. Shorter distances from human activity also reduce the species’ foraging efficiency due to increased vigilance behavior [47].

### Conservation implications

This is the first attempt to investigate the red-crowned crane—habitat relationship at multiple scales in the YRDNR. Our results suggest that a multi-scale approach can provide more comprehensive support than other methods for developing a protection strategy for the red-crowned crane. To increase the protection of the red-crowned crane, the focus of conservation effects should be directed toward landscape- and plot-level factors. In particular, it is critical to enhance the protection of seepweed tide flats, tamarisk-seepweed tidal flats, reed marshes and other natural wetlands, maintaining or expanding their existing habitat areas at the landscape scale. We also found that road density was negatively associated with crane occurrence. This finding suggests that road construction associated with oil wells and agricultural development has a negative effect on crane occurrence. Reconciling the contradictions between conservation and development is a key issue for local managers, as the YRDNR is also one of the most important regions of petroleum production in China.

The final autologistic model demonstrated the reliability of our predictions in terms of predictive capacity (CCR = 0.929, kappa value = 0.857, AUC value = 0.991). Our model showed that the southern part of the reserve made up most of the cranes’ suitable habitat and had better habitat quality than the northern part. This finding is not unexpected, as the southern part is located in the Yellow River estuary, where fresh water and sediment discharge carried by the Yellow River produce large amounts of suitable habitat. Furthermore, the southern wetland condition has improved since the initiation of a wetland restoration project in 2002 [25]. The northern part of the reserve has experienced drastic coastline erosion with the intrusion of seawater since the alteration of the river channel in 1976 [26]. This condition continues to cause the loss and degradation of crane habitat.

Our model also showed that there was a large protection gap for red-crowned cranes in the YRDNR accounting for 69.51% of the total suitable habitat. Almost all of the suitable crane habitat falls outside the core zone in the northern part and is mainly distributed in the experimental zone. This condition may reflect the fact that the functional zoning map of the YRDNR represents a trade-off between wetland habitat protection and oil field development, which has resulted in a number of crane habitats being distributed within oil-producing areas [25].

Therefore, we propose strengthening the protection of the red-crowned crane in three ways. First, managers should strengthen the restoration of degraded wetlands, especially in the northern part of the reserve; second, the functional zoning map and re-division of the core zone should be adjusted in the near future based on suitable crane habitat; and third, human disturbance should be strictly managed and effective measures implemented to minimize the negative effects of oil exploitation and road development in the YRDNR.
